# Ubiquitin receptors play redundant roles in the proteasomal degradation of the p53 repressor MDM2

**DOI:** 10.1002/1873-3468.14436

**Published:** 2022-07-21

**Authors:** Alison Sparks, Christopher J. Kelly, Mark K. Saville

**Affiliations:** ^1^ School of Medicine University of Dundee UK; ^2^ Institute of Infection, Immunity and Inflammation University of Glasgow UK; ^3^ Silver River Editing Dundee UK

**Keywords:** ADRM1/PSMD16/hRpn13, MDM2, p53/TP53, proteasomal ubiquitin receptor, redundancy, S5A/PSMD4/hRpn10

## Abstract

Much remains to be determined about the participation of ubiquitin receptors in proteasomal degradation and their potential as therapeutic targets. Suppression of the ubiquitin receptor S5A/PSMD4/hRpn10 alone stabilises p53/TP53 but not the key p53 repressor MDM2. Here, we observed S5A and the ubiquitin receptors ADRM1/PSMD16/hRpn13 and RAD23A and B functionally overlap in MDM2 degradation. We provide further evidence that degradation of only a subset of ubiquitinated proteins is sensitive to S5A knockdown because ubiquitin receptor redundancy is commonplace. p53 can be upregulated by S5A modulation while degradation of substrates with redundant receptors is maintained. Our observations and analysis of Cancer Dependency Map (DepMap) screens show S5A depletion/loss substantially reduces cancer cell line viability. This and selective S5A dependency of proteasomal substrates make S5A a target of interest for cancer therapy.

## Graphical abstract

Targeting of substrates to the proteasome involves ubiquitin‐binding receptors. Here, we show that ubiquitin receptors play redundant roles in degradation of the p53 repressor MDM2. In contrast, p53 degradation appears to be somewhat unusual in being markedly sensitive to suppression of the ubiquitin receptor S5A alone. We show that targeting S5A reduces cancer cell line viability and provide evidence that p53 contributes to this.

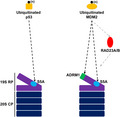

## Abbreviations

**CP**, core particle

**DepMap**, [Cancer] Dependency Map

**RP**, regulatory particle

**SRB**, sulphorhodamine B

**UBA**, ubiquitin‐associated

**UBL**, ubiquitin‐like

**UIM**, ubiquitin‐interacting motif

An essential role of covalent conjugation of ubiquitin to proteins is to mark them for degradation by the 26S proteasome. This commonly involves chains of ubiquitin cross‐linked through internal lysine residue 48, but other forms of ubiquitin modification can also target proteins to the proteasome [1]. Proteasomal substrates include critical cellular regulators like the tumour suppressor p53/TP53 and damaged or misfolded proteins. Inhibitors of the proteolytic activities of the proteasome, namely bortezomib, carfilzomib and ixazomib, have made a considerable impact on the treatment of multiple myeloma. However, resistance is a major problem and these inhibitors have been unsuccessful in treating solid tumours [2, 3, 4, 5, 6]. Study of the machinery involved in the recruitment of ubiquitinated substrates to the proteasome is important for understanding how protein degradation is mediated and controlled. It is also relevant to identifying and validating targets for cancer treatment, particularly given the therapeutic successes and limitations of currently used proteasome inhibitors.

The 26S proteasome is composed of two multiprotein complexes: the 20S core particle (CP), which carries out protein degradation, and the 19S regulatory particle (RP), which mediates substrate recruitment. The targeting of ubiquitinated substrates to the proteasome for degradation is facilitated by ubiquitin‐binding receptors [7, 8, 9, 10, 11]. Some receptors are intrinsic components of the 19S RP. These include S5A/PSMD4/hRpn10 and ADRM1/PSMD16/hRpn13, which have been shown to participate in substrate recruitment in mammalian cells [12, 13, 14, 15, 16, 17]. The simultaneous loss of S5A and ADRM1 is not sufficient to abolish ubiquitin binding to the proteasome showing there are additional ubiquitin receptors [17]. Rpn1 is a major ubiquitin receptor in the yeast 19S RP, and its human orthologue PSMD2 can also interact with ubiquitin [18, 19]. In addition, the 19S RP subunit SEM1/PSMD15/DSS1/SHFM1 may be involved in substrate recognition [20, 21]. There are also ubiquitin receptors that interact transiently with the proteasome and are thought to act as shuttles for ubiquitinated substrates. These include adaptor proteins such as RAD23A and B and UBQLN1 to 4 that contain proteasome‐binding ubiquitin‐like (UBL) domains and ubiquitin‐associated (UBA) domains that bind to ubiquitin [7, 9, 22, 23]. As well as interacting directly with substrate conjugated ubiquitin, S5A and ADRM1 can facilitate proteasomal delivery of substrates by complexing with the UBL domains of these adaptor proteins [13, 17, 24]. ZFAND5, which interacts with the proteasome and through its zinc finger A20 domain binds ubiquitin, has also been reported to be involved in substrate recruitment [25, 26]. SQSTM1/p62 is a ubiquitin receptor that participates in selective autophagy [23, 27]. In addition, SQSTM1 binds to the proteasome through S5A and targets substrates for proteasomal degradation [28, 29]. There is considerable ubiquitin receptor redundancy for protein degradation. This has been most extensively studied in yeast, where Rpn10, the orthologue of S5A, and Rpn13, the orthologue of ADRM1, have substantially overlapping functions in the recruitment of ubiquitinated proteins to the proteasome [13, 18, 30]. In addition, there is redundancy between Rpn10 and Rpn13 and yeast UBL‐UBA adaptor proteins, including Rad23, the orthologue of RAD23A/B and Dsk2, the orthologue of UBQLNs [13, 31, 32, 33, 34]. There is also evidence that S5A and ADRM1, to a high degree, play redundant roles in degrading ubiquitinated proteins in mammalian cells [17].

Ubiquitin‐dependent proteasomal degradation plays a critical role in determining the balance between p53 and MDM2 [35, 36, 37, 38, 39]. MDM2 participates in a negative feedback loop to limit the activity of p53. The *MDM2* gene is transcriptionally upregulated by p53. The binding of MDM2 to p53 directly represses p53's transcriptional activity [40, 41]. In addition, p53 is targeted for proteasomal degradation by MDM2‐mediated ubiquitination. MDM2 is also degraded by the proteasome: MDM2 can auto‐ubiquitinate, and it is a substrate for other ubiquitin E3 ligases [42]. Several studies have addressed the role of ubiquitin receptors in p53 degradation, but much remains to be learned and the pathway of proteasomal recruitment of MDM2 is unknown. We previously observed that the degradation of p53 but not MDM2 is inhibited by suppression of S5A alone and that p53 degradation requires the ubiquitin‐interacting motifs (UIMs) of S5A [15]. This is consistent with work showing that p53 is accumulated following knockdown of S5A in a range of cell types [43]. In HPV18‐positive cells, in which HPV protein E6 and cellular E6AP mediate p53 degradation rather than MDM2 [44], S5A knockdown also increases p53 levels [45]. RAD23A directly binds to MDM2, but the impact of RAD23A or B depletion on the level of p53 varies between studies and its effect on the stability of MDM2 has not been addressed [46, 47, 48]. UBQLN1 knockdown was reported to decrease p53 levels [49], while in HPV18‐positive cells, depletion of SHFM1 has been observed to elevate p53 expression [21].

This study further explored the role of intrinsic proteasomal ubiquitin receptors and ubiquitinated‐substrate shuttle proteins in the degradation of p53 and MDM2. We examined the effects of targeting individual ubiquitin receptors and receptors in combination. Suppression of S5A alone strongly attenuates p53 degradation. In contrast, individual knockdown of other receptors, including ADRM1 and the UBL‐UBA adaptor proteins RAD23A or B, did not affect p53 stability. MDM2 was not stabilised by targeting single ubiquitin receptors. However, MDM2 degradation was partially blocked by the simultaneous suppression of S5A and depletion of ADRM1 and further inhibited when RAD23A or B were also knocked down. This indicates ubiquitin receptors can function redundantly in the degradation of MDM2. Effects on the degradation of other proteasomal substrates and on the levels of cellular ubiquitin conjugates provide additional evidence that redundancy between S5A and ADRM1 is relatively common. Our observations and assessment of Cancer Dependency Map (DepMap) knockdown and knockout screens show depletion or loss of S5A is, however, sufficient to impact cancer cell line viability. Results are consistent with a contribution of wild‐type p53 to reducing cell viability following S5A suppression but show there are also other substrates involved.

## Materials and methods

### Cell culture

A375 melanoma‐derived cells were cultured in Dulbecco's modified Eagle's medium, and HCTT16 colon cancer‐derived cells were cultured in McCoy's 5A medium. Both media were supplemented with 10% fetal bovine serum and 50 μg·mL^−1^ gentamycin. Cells were grown at 37 °C, 5% CO_2_ in a humidified atmosphere. HCT116^Ex2p53−/−^ cells, where exon two of the *p53* gene is deleted, were previously derived by homologous recombination [50]. For western blot analysis, cells were seeded onto six‐well plates: A375 (0.6 × 10^5^ cells per well), HCT116^p53+/+^ (1.5 × 10^5^ cells per well). For viability assays, cells were seeded onto 96‐well plates: A375 (500 cells per well), HCT116^p53+/+^ (2000 cells per well) and HCT116^Ex2p53−/−^ (2500 cells per well). Bortezomib, LC Laboratories (Woburn, MA, USA), and cycloheximide, Merck Group (Darmstadt, Germany), were added for the indicated times before harvesting.

### siRNA transfection

Transfection of siRNAs was carried out according to the manufacturer's instructions using Lipofectamine RNAiMAX transfection reagent, Thermo Fisher Scientific (Waltham, MA, USA). Unless otherwise indicated, siRNAs were introduced into cells by forward transfection and individual siRNAs were used at a final concentration of 10 nm. For experiments involving transfections with siRNA combinations, where appropriate, non‐targeting siRNA was included to give a uniform total siRNA concentration. Dharmacon ON‐TARGETplus modified siRNAs, Horizon Discovery (Cambridge, UK), were used throughout: non‐targeting control, D‐001810‐01; S5A(a), J‐011365‐05; S5A(b), J‐011365‐07; ADRM1(a), J‐012340‐10; ADRM1(b), J‐012340‐11; RAD23A(a), J‐005231‐05; RAD23A(b), J‐005231‐06; RAD23A(c), J‐005231‐07; RAD23B(a), J‐011759‐05; RAD23B(b), J‐011759‐06; RAD23B(c), J‐011759‐07 and the additional siRNAs listed in Fig. S1.

### Adenovirus transduction

Adenoviruses were constructed using the AdEasy system, Agilent Technologies (Santa Clara, CA, USA) [51]. A control adenovirus was used that expresses GFP alone, along with an adenovirus expressing GFP and C‐terminally HA‐tagged S5AΔUIM (residues 1–195 of wild‐type S5A that lacks both UIMs). Twenty‐four hours after reverse transfection with siRNAs, the normal growth medium was replaced with medium containing 2% fetal bovine serum and cells were infected with adenovirus at a multiplicity of infection of 1000. After 4 h, cells were washed twice in PBS, returned to normal growth medium and harvested 40 h after infection.

### Western blotting

Cells were extracted by direct lysis into SDS‐urea electrophoresis sample buffer, and western blotting was carried out as previously described [52]. To expose epitopes, membranes to be probed for ubiquitin were boiled in de‐ionised water for 30 min before blocking. The primary antibodies used were p53 (SAPU), Scottish Antibody Production Unit (Carluke, UK); MDM2 (4B2), Moravian‐Biotechnology (Brno, Czech Republic); S5A (14899‐1‐AP), Proteintech Group (Rosemont, IL, USA); ADRM1 (ab56852), RAD23A (EPR4817, ab108591), RAD23B (ab3835), HA tag (HA.C5, ab18181), NOXA (114C307, ab13654) and beta‐actin (ab8226), Abcam (Cambridge, UK); Ubiquitin (P4D1‐A11, 05‐944), p21 (EA10, OP64/Ab‐1) and GFP (mixture of clones 7.1 and 13.1, 11814460001), Merck Group; c‐MYC (9E10), prepared in house and MCL‐1 (D35A5, #5453), Cell Signaling Technology (Danvers, MA, USA). Suitable exposures of western blots were quantified by densitometry using Quantity One, Bio‐Rad (Hercules, CA, USA). SeeBlue Plus2 protein standards were obtained from Thermo Fisher Scientific.

### Viability assays

Live cell number and cell death were analysed according to the manufacturers' instructions with an IncuCyte ZOOM real‐time imager, Essen BioScience (Ann Arbor, MI, USA), using the CellTox Green Cytotoxicity Assay, Promega (Madison, WI, USA). Sulphorhodamine B (SRB) assays were carried out as previously described [53]. Cells were fixed in 10% TCA for 60 min at 4 °C and then washed five times with water. Following staining in 0.4% (w/v) SRB in 1% acetic acid for 30 min, plates were washed five times with 1% acetic acid. Bound stain was solubilised in 10 mm Tris base, and the absorbance was measured at 515 nm.

### Cancer DepMap analysis

The DepMap Portal was used to assess data from large‐scale knockdown and knockout viability screens involving a comprehensive panel of cancer cell lines [54]. The RNAi screens were carried out by the Broad Institute (Project Achilles) [55, 56], Novartis (Project DRIVE) [57] and Marcotte et al. (Marcotte) [58]. The CRISPR screens were carried out by the Wellcome Sanger Institute (Project Score) [59, 60] and the Broad Institute (DepMap Public) [61, 62, 63, 64]. The *p53* mutational status of cell lines was given by Mutation 22Q1 Public [54]. The following data sets were used: Achilles, DRIVE and Marcotte RNAi screens [65], Wellcome Sanger Institute Project Score [66] and Broad Institute DepMap Public 22Q1 [67].

Screens involved the transduction of cancer cell lines with viruses expressing a pooled library of shRNAs or sgRNAs, with infection rates such that individual cells received a maximum of a single shRNA or sgRNA. Multiple different shRNA or sgRNAs were used per gene. Cells were allowed to proliferate, and the representation of shRNA or sgRNAs, predominantly determined by sequencing, was used to infer the effect on viability. The data had been processed to yield a gene effect score using algorithms that address several issues. DEMETER2, which had been used to process the results from RNAi screens, addresses parameters including variable shRNA efficacy, seed region‐based off‐target effects and variations in the strength of gene knockdown between cell lines [56]. Chronos, which had been used to process the results from CRISPR screens, addresses parameters including sgRNA efficacy, heterogeneity in the effects of DNA cutting on gene function, biases due to gene copy number differences and variations in cell line growth rates and is relatively robust to sgRNA off‐target effects [63].

We show the percentage of cell lines classified as dependent on a gene for maintaining viability. This is based on dependency probabilities, which had been calculated as described using gene effect scores [62]. In each cell line, the score for a given gene had been compared with the scores for non‐essential and essential genes. A line had been classified as dependent on a gene if the score for the gene is likely to have fallen into the range of scores for essential genes. We also show the gene effect scores. A negative gene effect score indicates a decrease in viability and a positive gene effect score an increase in growth. The results had been offset and scaled such that 0 is the gene effect score for a non‐essential gene and a gene effect score of −1 is the median of all common essential genes.

### Statistical analysis

One‐way ANOVA and Bonferroni *post hoc* tests and two‐tailed *t*‐tests were carried out using graphpad prism, graphpad Software (San Diego, CA, USA). Pearson correlation coefficients and two‐tailed *P* values were obtained using the DepMap Portal [54] or using graphpad prism.

## Results

### While the degradation of p53 is strongly sensitive to depletion of S5A alone, ubiquitin receptors have overlapping functions in the degradation of MDM2

**Fig. 1:**
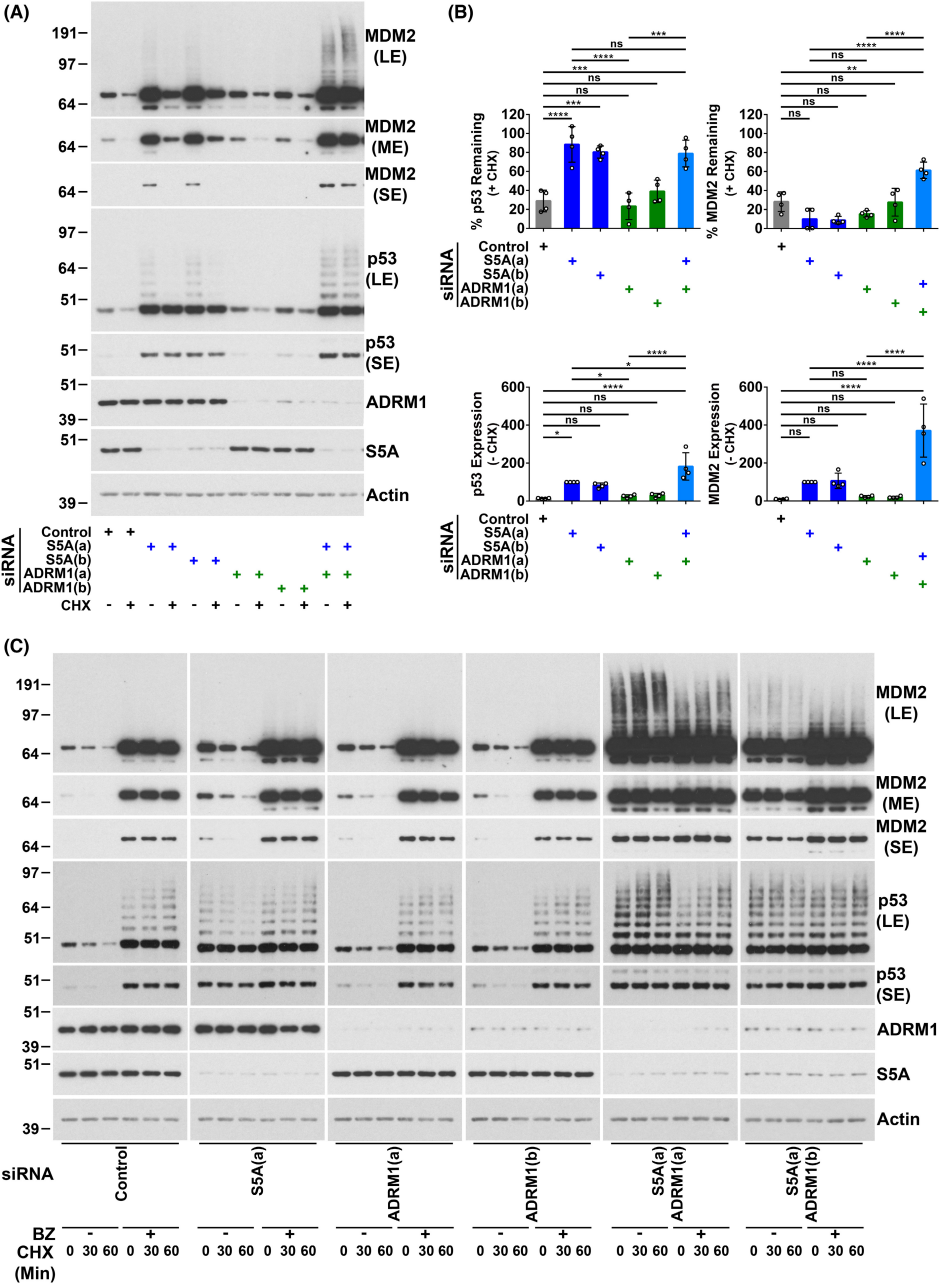
Knockdown of S5A alone is sufficient to stabilise p53, while depletion of both S5A and ADRM1 is required to inhibit MDM2 degradation. A375 cells were harvested 48 h after transfection with the indicated siRNAs and analysed by western blotting. A non‐targeting siRNA (Control) was used. S5A and ADRM1 siRNA (A) and (B) are complementary to different target sequences. Cycloheximide (CHX; 20 μg·mL^−1^) was added for 60 min unless otherwise indicated to assess protein stability. (A) Representative western blots. Short (SE), medium (ME) and long (LE) exposures are included. (B) Western blot results expressed as the percentage remaining in cycloheximide‐treated cells (upper panels: protein stability) or, in the absence of cycloheximide, as a percentage of the level in cells transfected with siRNA S5A (A) (lower panels: protein expression). The mean and SD are shown, and individual data points from four experiments are plotted, ns, not significant, *P < 0.05, **P < 0.01, ***P < 0.001, ****P < 0.0001 using one‐way ANOVA and Bonferroni *post hoc* test. (C) Comparison of the effects on p53 and MDM2 of combined S5A and ADRM1 depletion and bortezomib‐mediated proteasome inhibition (BZ; 100 nm for 5 h). Representative western blots. Short (SE), medium (ME) and long (LE) exposures are included.

**Fig. 2:**
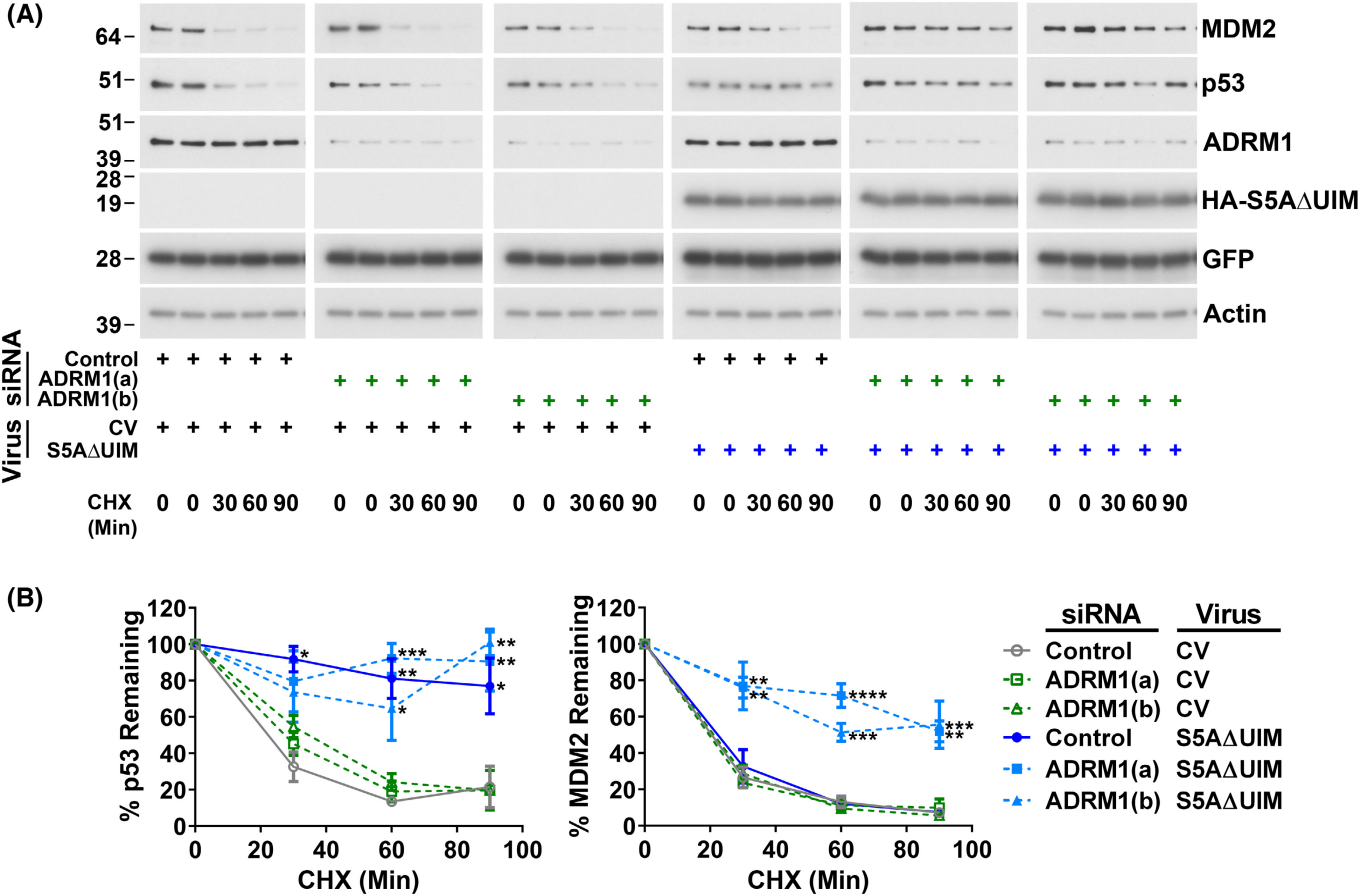
Ectopic expression of S5AΔUIM is sufficient to inhibit the degradation of p53, but attenuation of MDM2 degradation requires the simultaneous expression of S5AΔUIM and depletion of ADRM1. A375 cells were reverse transfected with non‐targeting siRNA (Control) or siRNAs ADRM1 (A) or (B) and 24 h later transduced with a control adenovirus (CV) expressing GFP alone or an adenovirus expressing GFP and an HA‐tagged deletion of S5A lacking UIMs (S5AΔUIM). Cycloheximide (CHX; 20 μg·mL^−1^) was added for the indicated times to assess protein stability. Cells were harvested 40 h after infection and analysed by western blotting. (A) Representative western blots. To allow direct comparison of protein stability, different exposures of MDM2 and p53 blots are shown, so that band intensities in the absence of cycloheximide are approximately matched. Duplicate 0‐min technical replicates were included. (B) Western blot results expressed as the percentage remaining in cycloheximide‐treated cells (protein stability). The mean and SEM are shown (*n* = 3); all significant differences from cells transfected with control siRNA and transduced with control adenovirus are indicated, *P < 0.05, **P < 0.01, ***P < 0.001, ****P < 0.0001 using one‐way ANOVA and Bonferroni *post hoc* test.

We compared the effects on wild‐type p53 and MDM2 of siRNA‐mediated knockdown of the intrinsic proteasomal ubiquitin receptors S5A and ADRM1 alone and in combination in A375 melanoma cells. To assess protein stability, cycloheximide was added before harvesting for analysis by western blotting. Depletion or loss of S5A or ADRM1 does not grossly impact on proteasome assembly [15, 16, 17, 34, 68, 69, 70]. It has been suggested this is because S5A and ADRM1 are at the periphery of the 19S RP [19, 71, 72, 73]. In line with our previous observations [15], knockdown of S5A stabilised p53 but not MDM2 and caused the accumulation of p53‐ubiquitin conjugates (Fig. 1). MDM2 protein levels were increased by S5A knockdown. This is a consequence of the upregulation of *MDM2* mRNA expression due to elevated p53 transcriptional activity [15]. Knockdown of ADRM1 alone was not observed to affect the stability of p53 or MDM2. However, the simultaneous depletion of S5A and ADRM1 substantially blocked MDM2 degradation (Fig. 1). The knockdown of S5A and ADRM1 together also resulted in the accumulation of high molecular weight MDM2 conjugates (Fig. 1A,C). This is consistent with attenuated degradation of ubiquitinated MDM2. Mean p53 and MDM2 protein levels were slightly higher in cells depleted of ADRM1. However, this did not reach significance. There was a limited further effect on p53 levels from combining ADRM1 knockdown with S5A depletion, but no increase in p53 stability was detected. This may be due to a small additional change being difficult to measure because of the strong impact of targeting S5A. Inhibition of the 20S CP using bortezomib confirmed that the proteasome continued to degrade p53 in cells depleted of ADRM1 and MDM2 in cells depleted of S5A or ADRM1 (Fig. 1C). In contrast, the modest further effects of bortezomib showed that knockdown of S5A strongly inhibited proteasomal degradation of p53 and that combined knockdown of S5A and ADRM1 substantially reduced proteasomal degradation of MDM2. A deletion mutant of S5A (S5AΔUIM) lacking its two UIMs but retaining its proteasome‐binding domain was also used to investigate the involvement of S5A. In agreement with our previous findings [15], ectopic expression of S5AΔUIM inhibited p53 degradation without influencing the stability of MDM2 (Fig. 2). This is consistent with the incorporation of S5AΔUIM into the proteasome competitively with endogenous S5A and consequent interference with functions that are dependent on endogenous S5A's ubiquitin‐binding activity [15, 74]. Here, we observed that the degradation of MDM2 was inhibited by ectopic expression of S5AΔUIM in combination with ADRM1 knockdown (Fig. 2). This indicates the UIMs of S5A participate in MDM2 degradation. Overall, these approaches confirm that S5A has a major non‐redundant role in the proteasomal degradation of p53 but show that S5A and ADRM1 play redundant roles in the proteasomal degradation of MDM2.

**Fig. 3:**
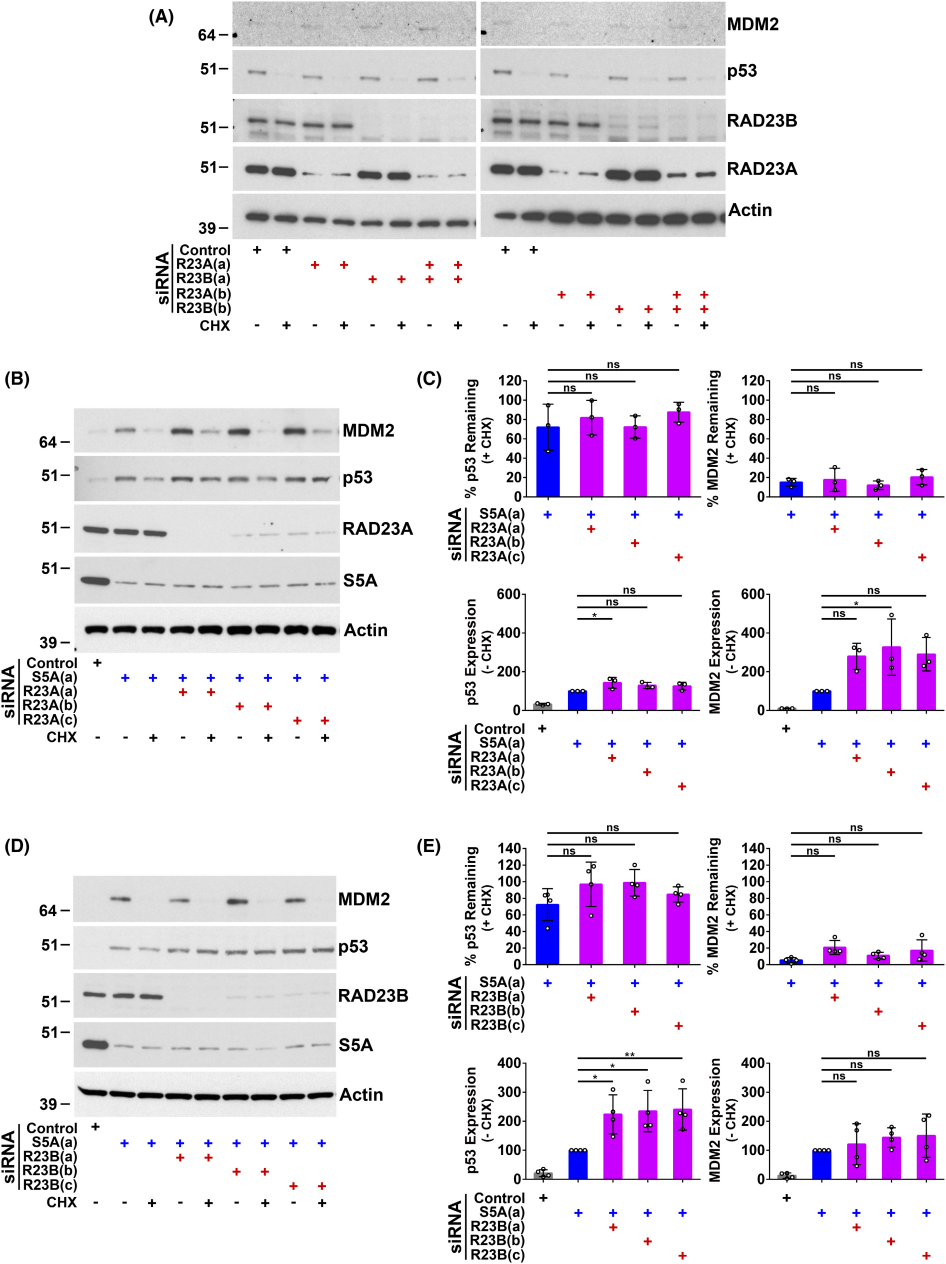
Depletion of RAD23A and B alone or in combination with S5A does not significantly increase the stability of p53 or MDM2. A375 cells were harvested 48 h after siRNA transfection and analysed by western blotting. RAD23A and B siRNAs (A) (B) and (C) are complementary to different target sequences. Cells were incubated with cycloheximide (CHX; 20 μg·mL^−1^) for the final 60 min to assess protein stability. (A) Knockdown of RAD23A and B alone or in combination did not substantially influence the stability of p53 or MDM2. Representative western blots are shown. (B and C) Combining knockdown of RAD23A with S5A depletion did not increase the stability of p53 or MDM2. (B) Representative western blots. (C) Western blot results expressed as the percentage remaining in cycloheximide‐treated cells (upper panels: protein stability) or, in the absence of cycloheximide, as a percentage of the level in cells transfected with siRNA S5A(A) (lower panels: protein expression). The mean and SD are shown, and individual data points from three experiments are plotted, ns, not significant, *P < 0.05 using one‐way ANOVA and Bonferroni *post hoc* test. (D and E) Combining knockdown of RAD23B with S5A depletion did not significantly increase the stability of p53 or MDM2. (D) Representative western blots. (E) Western blot results expressed as the percentage remaining in cycloheximide‐treated cells (upper panels: protein stability) or, in the absence of cycloheximide, as a percentage of the level in cells transfected with siRNA S5A(A) (lower panels: protein expression). The mean and SD are shown, and individual data points from four experiments are plotted, ns, not significant, *P < 0.05, **P < 0.01 using one‐way ANOVA and Bonferroni *post hoc* test.

**Fig. 4:**
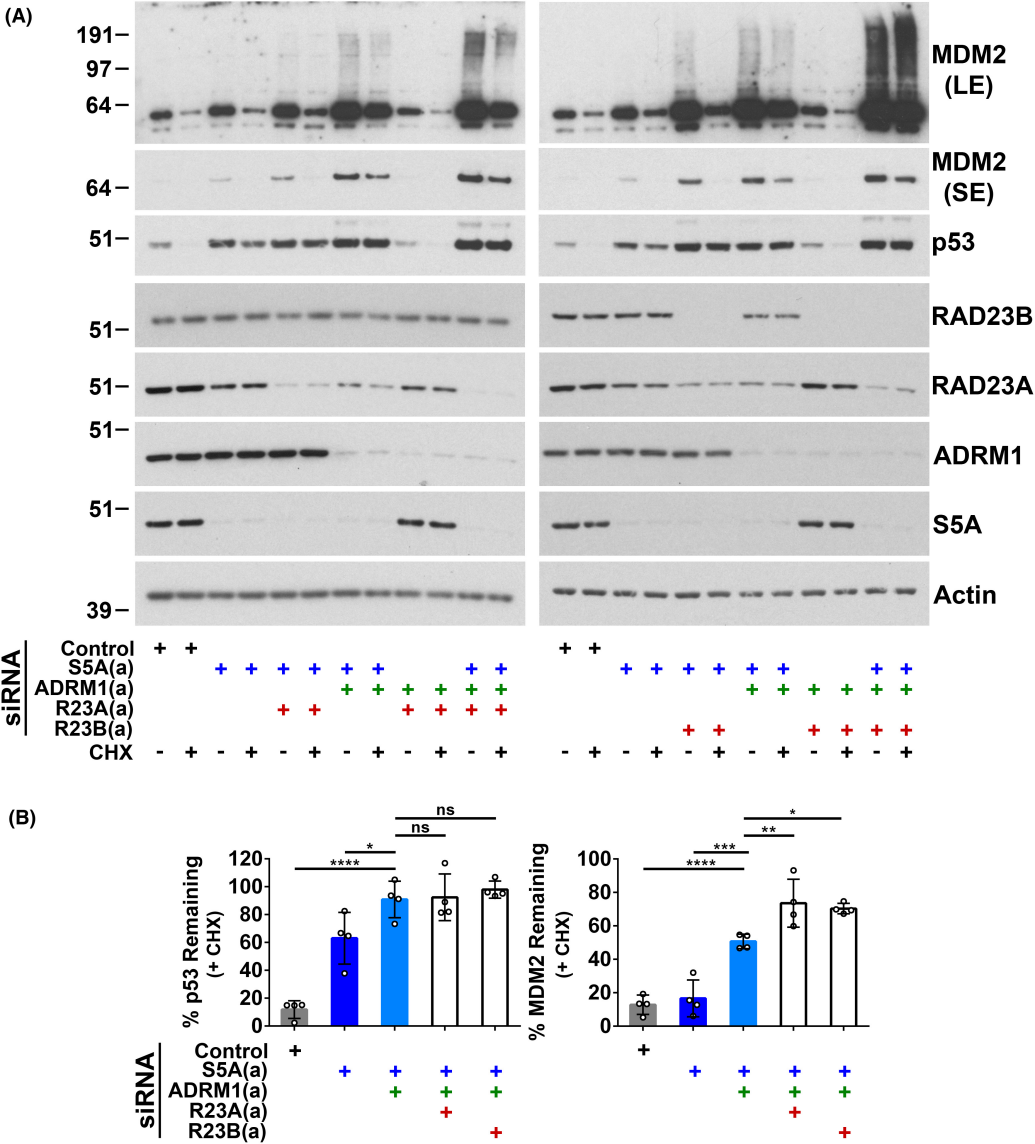
RAD23A and B play redundant roles with S5A and ADRM1 in the degradation of MDM2. A375 cells were harvested 48 h after siRNA transfection and analysed by western blotting. Cells were incubated with cycloheximide (CHX; 20 μg·mL^−1^) for the final 60 min to assess protein stability. Knockdown of RAD23A or B in cells where both S5A and ADRM1 were also depleted further stabilised MDM2. (A) Representative western blots. Short exposures (SE) and long exposures (LE) are included for MDM2. (B) Western blot results expressed as the percentage remaining in cycloheximide‐treated cells (protein stability). The mean and SD are shown and individual data points from four experiments are plotted, ns, not significant, *P < 0.05, **P < 0.01, ***P < 0.001, ****P < 0.0001 using one‐way ANOVA and Bonferroni *post hoc* test.

Next, the participation of additional ubiquitin receptors, including shuttle proteins, in the degradation of p53 and MDM2 was investigated. The strongest prior evidence was for an influence of the UBL‐UBA shuttle proteins RAD23A and B on MDM2‐mediated degradation of p53 [46, 47, 48]. In A375 cells, knockdown of RAD23A and B alone or the simultaneous depletion of RAD23A and B did not affect the stability of p53 or MDM2 (Fig. 3A). Using pools of siRNA (Fig. S1), we also screened to look for the involvement in the degradation of p53 or MDM2 of 14 additional ubiquitin‐binding proteins, including SEM1, UBQLN1 to 4, DDI1 and 2, ZFAND5 and SQSTM1. No effect on p53 or MDM2 stability was detected following the transfection of A375 cells with these receptor‐targeting siRNA pools (Fig. S2). We also determined the impact of depleting RAD23A and B in combination with S5A. Knockdown of RAD23A had little or no further effect on p53 levels or degradation in cells in which S5A was depleted (Fig. 3B,C). In addition, no significant MDM2 stabilisation was observed with S5A knockdown combined with knockdown of RAD23A or B (Fig. 3B–E). The simultaneous knockdown of RAD23B and S5A increased p53 expression compared with depletion of S5A alone (Fig. 3D,E). There was a tendency for the depletion of RAD23B in combination with S5A to increase p53 stability further, but this failed to reach significance (Fig. 3E, upper left panel). Again, this may be due to modest changes that are challenging to measure because of strong p53 stabilisation following S5A knockdown. RAD23A or B knockdown was also combined with simultaneous depletion of S5A and ADRM1. As previously (Fig. 1), the knockdown of S5A and ADRM1 together partially inhibited MDM2 degradation. Incorporating RAD23A or RAD23B knockdown caused a significant additional increase in MDM2 stability, associated with a greater accumulation of high molecular weight MDM2 conjugates (Fig. 4). These data indicate that RAD23A/B can also contribute to MDM2 degradation and suggest that there are functionally overlapping independent pathways for MDM2 degradation involving S5A, ADRM1 and RAD23A/B.

### S5A and ADRM1 play partially redundant roles in general protein degradation; however, S5A depletion or loss can substantially reduce cancer cell viability

**Fig. 5:**
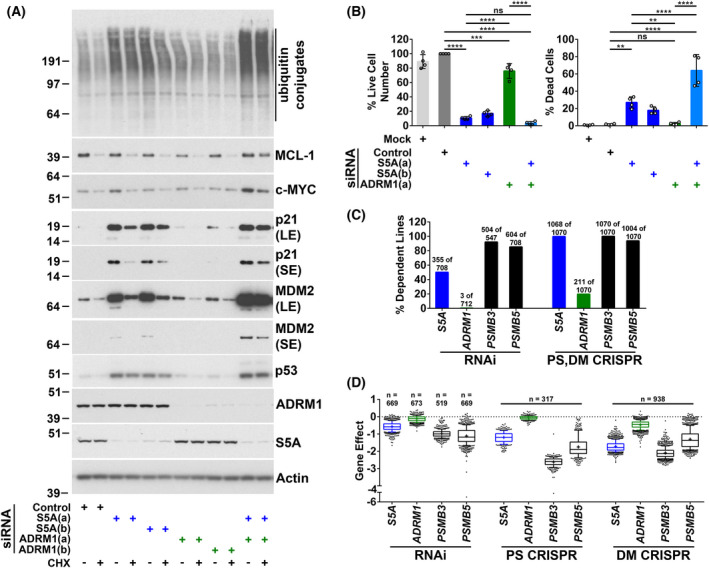
S5A and ADRM1 play partially redundant roles in general protein degradation, but S5A makes a substantial contribution to maintaining the viability of cancer cells. (A) The simultaneous knockdown of S5A and ADRM1 had a greater effect on the accumulation of ubiquitin conjugates and, except for p53, the stability of proteasomal substrates tested. A375 cells were harvested 48 h after siRNA transfection. Cells were incubated with cycloheximide (CHX; 20 μg·mL^−1^) for 60 min to assess protein stability. Representative western blots are shown. Short exposures (SE) and long exposures (LE) are included. (B) While the combined depletion of S5A and ADRM1 induced the greatest level of cell death, targeting S5A but not ADRM1 alone was sufficient to reduce cell viability markedly. A375 cells were mock‐transfected (Mock) or transfected with the indicated siRNAs. The number of live cells expressed as a percentage of non‐targeting siRNA transfected cells and the percentage of dead cells was assessed 96 h after siRNA reverse transfection by real‐time imaging. The mean and SD are shown and individual data points from four experiments are plotted, ns, not significant, **P < 0.01, ***P < 0.001, ****P < 0.0001 using one‐way ANOVA and Bonferroni *post hoc* test. (C and D) Targeting S5A reduces the viability of cancer cell lines. (C) Dependency probability analysis of Achilles, DRIVE and Marcotte knockdown (RNAi) and Project Score and DepMap 22Q1 Public knockout (PS, DM CRISPR) cancer cell line screens. The results are expressed as a percentage of the cancer cell lines classified as being dependent for viability on *S5A*, *ADRM1* and, for comparison, the genes encoding 20S CP subunits PSMB3 and 5. The number of dependent cell lines and the total number of cell lines screened are shown. (D) DepMap portal analysis of the influence on viability (gene effect) of targeting the indicated gene in Achilles, DRIVE and Marcotte knockdown screens (RNAi) and Project Score (PS CRISPR) and DepMap 22Q1 Public (DM CRISPR) knockout screens. Box and whisker plots are shown. A negative gene effect score indicates a decrease in viability. The boxes extend from the 25^th^ to 75^th^ percentiles. The whiskers extend to the 10^th^ and 90^th^ percentiles. The median (line) and mean (+) are shown.

There can be a high degree of ubiquitin receptor redundancy for protein degradation. This has been observed in yeast [13, 18, 30, 31, 32, 33, 34], and there is also evidence for considerable functional overlap between S5A and ADRM1 in mammalian cells [17]. To examine this, we determined the effect of ubiquitin receptor knockdown on the degradation of additional proteasomal substrates (Fig. S3A) and on the general pattern of ubiquitination. The degradation of all other substrates investigated—p21, c‐MYC and MCL‐1—was not substantially attenuated by siRNA‐mediated depletion of S5A or ADRM1 alone but was inhibited by the simultaneous knockdown of both ubiquitin receptors (Fig. 5A). As for MDM2, the increase in p21 levels following S5A knockdown is due to increased mRNA expression resulting from the upregulation of p53 transcriptional activity [15]. Targeting ADRM1 had little effect on the level of ubiquitin conjugates. In line with previous observations, S5A knockdown increased the level of ubiquitinated proteins to some extent [15, 16], but the greatest accumulation of conjugates was observed with combined knockdown of S5A and ADRM1 (Fig. 5A). These results provide further evidence that it is common for S5A and ADRM1 to play overlapping roles in protein degradation as observed for MDM2 and suggest p53 stability may be somewhat unusual in being strongly sensitive to suppression of S5A alone.

The proteasome is required for cell viability, but in some organisms, the loss of ubiquitin receptors, including orthologues of S5A [70, 75, 76, 77] or ADRM1 [13, 78], has a minimal impact on viability. Receptor redundancy can contribute to sustaining viability [18, 31, 32, 33]. In mice, loss of S5A or deletion of its UIMs is embryonic lethal [74] and deletion of ADRM1 can cause neonatal death [17]. However, liver‐specific postnatal deletion of ADRM1 or the UIMs of S5A does not affect cell viability [17, 74]. We assessed the roles of S5A and ADRM1 in maintaining cancer cell line viability. A375 cells are killed by inhibition of the proteolytic activity of the proteasome (Fig. S3B). In these cells, depletion of ADRM1 caused only a modest reduction in viability, while S5A knockdown led to a robust decrease in viability, associated with an increase in death (Fig. 5B). The combined knockdown of S5A and ADRM1 resulted in more cell death than depletion of either receptor alone. The Cancer DepMap Portal facilitates the evaluation of data from large‐scale RNAi and CRISPR screens in hundreds of cancer cell lines [54]. DepMap dependency probability analysis aims to identify genes that are essential for the viability of each cell line [62]. In RNAi screens, this analysis classified *S5A* as being essential in half of the cancer cell lines tested, while *ADRM1* was scored as being essential in very few cell lines (Fig. 5C). In CRISPR screens, *S5A* was classified as essential in almost all cancer cell lines employed, while *ADRM1* was scored as being essential in around 20% of lines. The impact of targeting the genes encoding the 20S CP subunits PSMB3/β3 and PSMB5/β5 is shown for comparison. Depletion of these subunits interferes with the assembly of the proteasome [79]. In both knockdown and knockout screens, these genes were classified as essential in a high proportion of cell lines. In line with the dependency probability analysis, negative gene effect scores, indicating reduced viability on gene suppression, were generally larger for *S5A* than *ADRM1* in RNAi and CRISPR screens (Fig. 5D). The gene effect scores indicate the reduction in viability caused by targeting S5A was generally somewhat less than that resulting from targeting the 20S CP subunits PSMB3 and PSMB5 in RNAi screens [55, 56, 57, 58] and Wellcome Sanger Institute CRISPR screens (Project Score) [59, 60, 63]. However, in Broad Institute CRISPR screens (DepMap 22Q1 Public) [61, 62, 63, 64], the gene effect scores were similar. Furthermore, a larger impact of ADRM1 loss was observed in the Broad Institute screens than in the Wellcome Sanger Institute screens. There are several differences between these two knockout screens [80] including the time after virus transduction of sgRNAs at which viability was assessed: 14 days for Wellcome Sanger Institute screens and 21 days for Broad Institute screens. Overall DepMap screens indicate that targeting S5A can substantially reduce cancer cell line viability. In addition, ADRM1 loss can have a marked effect on viability in a subset of cancer lines.

### Evidence for an involvement of wild‐type p53 in the effects of S5A suppression on cancer cell line viability

**Fig. 6:**
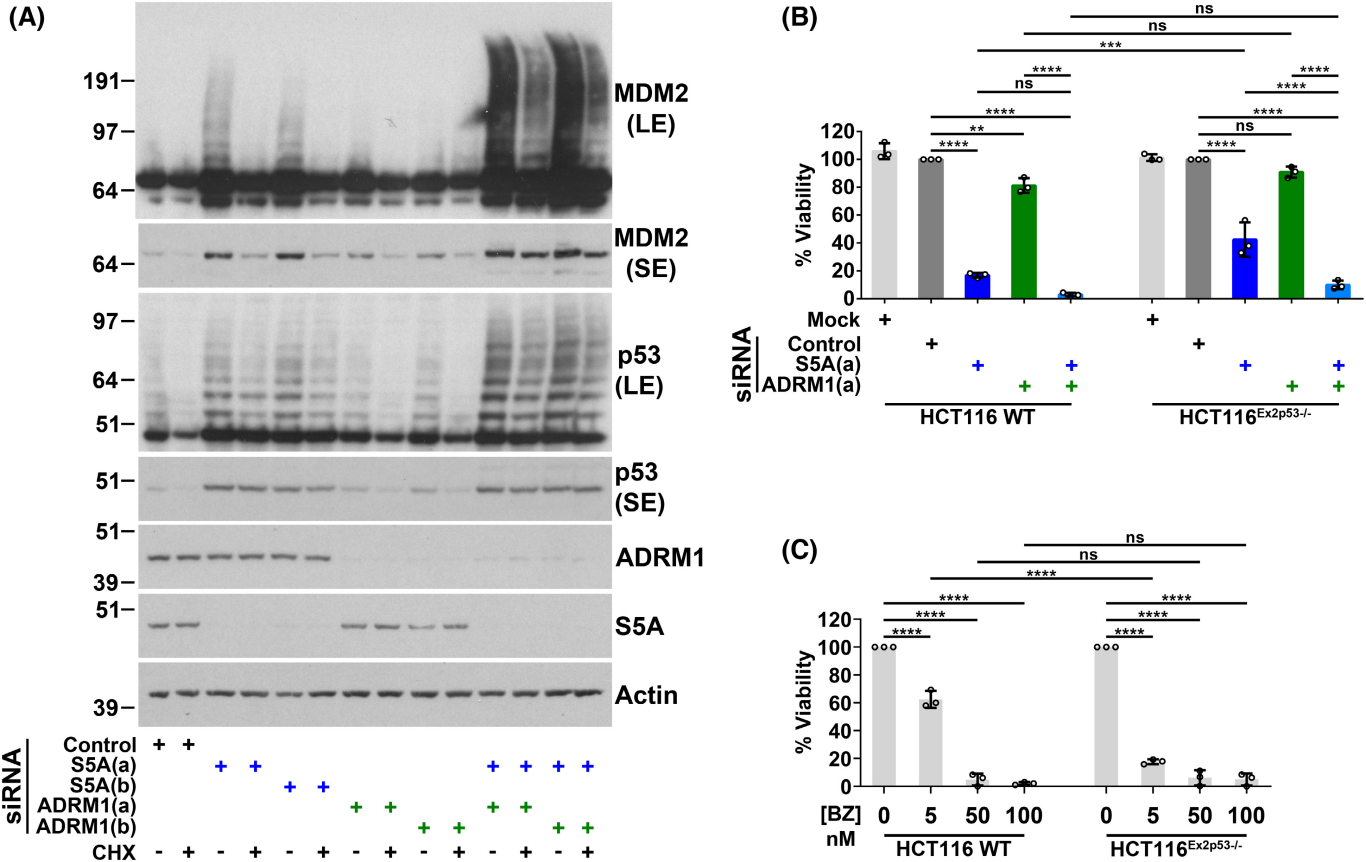
The reduction in viability caused by depleting S5A, but not other methods of targeting the proteasome, is greater in HCT116 cells with full‐length p53. (A) In HCT116 cells, the degradation of p53 was inhibited by depletion of S5A, while inhibition of MDM2 degradation required the knockdown of both S5A and ADRM1. HCT116 cells with wild‐type *p53* were harvested 48 h after transfection with the indicated siRNAs. Cycloheximide (CHX; 20 μg·mL^−1^) was added for 60 min to assess protein stability. Representative western blots are shown. For p53 and MDM2 short and long exposures are included (SE and LE, respectively). (B) In HCT116 cells, loss of full‐length p53 partially attenuated the decrease in viability caused by S5A knockdown, but it did not significantly influence the reduction in viability due to the simultaneous depletion of S5A and ADRM1. The viability of HCT116 cells with wild‐type *p53* (HCT116 WT) and HCT116 cells where exon two of the *p53* gene was deleted (HCT116^Ex2p53−/−^) was assessed 96 h after siRNA reverse transfection using the SRB assay. The mean and SD are shown and individual data points from three experiments are plotted, ns, not significant, **P < 0.01, ***P < 0.001, ****P < 0.0001 using one‐way ANOVA and Bonferroni *post hoc* test. (C) In HCT116 cells, loss of full‐length p53 increased sensitivity to submaximal concentrations of bortezomib but did not influence the decrease in viability observed at higher concentrations of this proteasome inhibitor. HCT116 WT and HCT116^Ex2p53−/−^ cells were incubated with the indicated concentration of bortezomib (BZ) and viability was assessed after 72 h using the SRB assay. The mean and SD are shown and individual data points from three experiments are plotted, ns, not significant, ****P < 0.0001 using one‐way ANOVA and Bonferroni *post hoc* test.

The p53‐dependency of the reduction in cell viability caused by ubiquitin receptor depletion was investigated by comparing responses in wild‐type *p53* HCT116^p53+/+^ cells and HCT116 cells lacking full‐length p53 due to deletion of exon two of the *p53* gene (HCT116^Ex2p53−/−^) [50]. In HCT116^p53+/+^ cells, as in A375 cells, depletion of S5A stabilised p53, but knockdown of both S5A and ADRM1 was required to inhibit MDM2 degradation (Fig. 6A). The viability of the HCT116 lines was assessed 4 days after siRNA transfection with S5A and ADRM1 siRNAs. At that time, control siRNA transfected HCT116^p53+/+^ and HCT116^Ex2p53−/−^ cells had undergone a similar level of growth: 4.1 (SD = 0.67) and 3.8 (SD = 0.093) doublings, respectively. S5A knockdown caused a substantial decrease in cell viability, and in agreement with previous observations [15], this was significantly greater in HCT116^p53+/+^ cells (Fig. 6B). Depletion of ADRM1 alone had little or no effect on the viability of HCT116 cells, but the combined knockdown of S5A and ADRM1 caused a larger reduction in viability than targeting the individual ubiquitin receptors. There was no significant difference in the effect on cell viability of simultaneous knockdown of these ubiquitin receptors in HCT116^p53+/+^ and HCT116^Ex2p53−/−^ cells. These results indicate that p53 can contribute to the decrease in viability resulting from S5A knockdown. Consistent with the stabilisation of a broader range of substrates, including MDM2, which can repress p53 by direct binding, the decrease in viability caused by the simultaneous depletion of S5A and ADRM1 was full‐length p53 independent. The robust reduction in viability caused by higher concentrations of bortezomib was similarly not influenced by p53 loss (Fig. 6C). Interestingly, at a submaximal concentration of bortezomib, we observed a greater effect on viability in HCT116^Ex2p53−/−^ cells indicating a protective role of full‐length p53. These results show there can be mechanistic differences underlying the effects on cell viability of depleting S5A and other ways of targeting the proteasome.

**Fig. 7:**
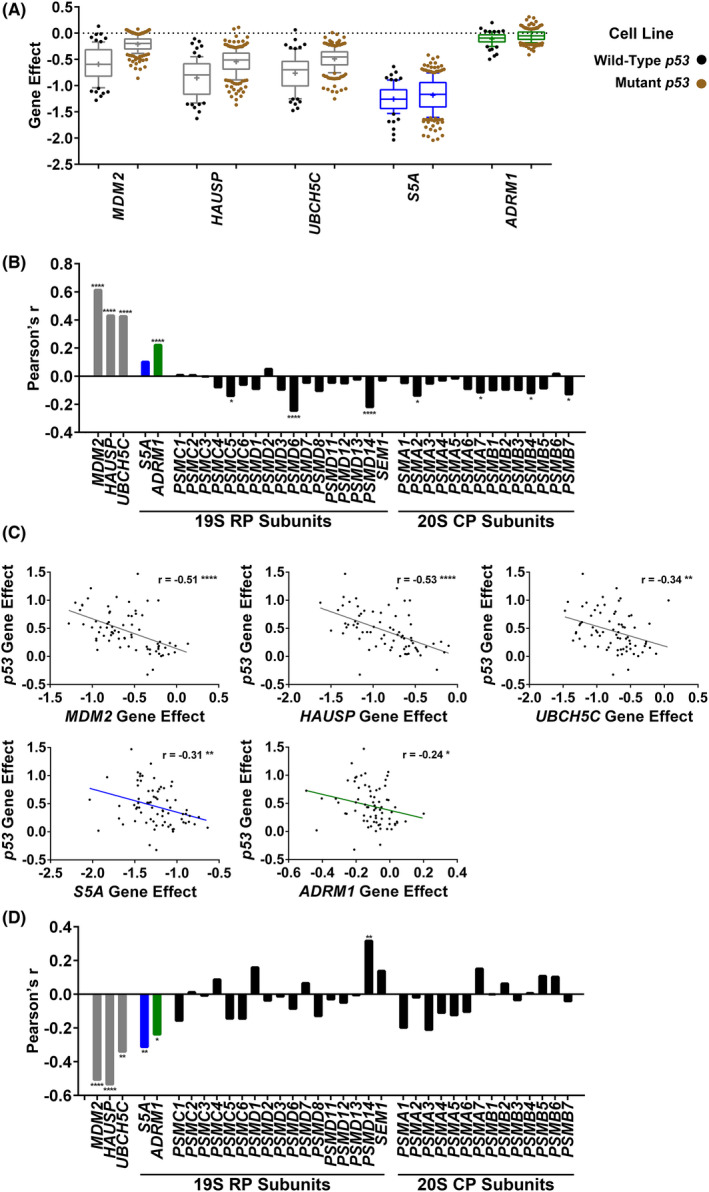
The effect of S5A knockout on viability is not strongly influenced overall by *p53* mutation; however, targeting S5A has a greater effect on viability in cell lines where wild‐type p53 is more active in limiting growth. (A and B) There is, at best, a weak correlation between the *p53* mutational status and the reduction in viability caused by targeting *S5A*. DepMap portal analysis of Project Score knockout screens in cancer cell lines. (A) The gene effect scores in cell lines with wild‐type *p53* (*n* = 72) and cell lines with *p53* mutation (*n* = 245) for *S5A* and *ADRM1* and, for comparison, genes encoding the wild‐type p53 repressors MDM2, HAUSP and UBCH5C. Box and whisker plots are shown. The boxes extend from the 25^th^ to 75^th^ percentiles. The whiskers extend to the 10^th^ and 90^th^ percentiles. The median (line) and mean (+) are shown. (B) The Pearson correlation coefficients (Pearson's *r*) for *p53* mutation and the gene effect scores for *S5A* and *ADRM1* and the genes encoding the indicated wild‐type p53 repressors and proteasome subunits (72 wild‐type *p53* cell lines and 245 mutant *p53* cell lines). All significant correlations are indicated, *P < 0.05, ****P < 0.0001 (two‐tailed). (C and D) Targeting S5A, but not proteasome subunits in general, has a greater effect on viability in cell lines where wild‐type p53 is more growth limiting. DepMap portal analysis of the correlation between the gene effect scores in cell lines with wild‐type *p53* for the indicated genes and *p53* in Project Score CRISPR knockout screens. (C) DepMap gene effect scores in wild‐type *p53* cell lines (*n* = 72) for the indicated genes plotted against the gene effect scores for *p53*. A negative gene effect score indicates a decrease in viability and a positive gene effect score an increase in growth. The Pearson's correlation coefficients are shown (*r*), *P < 0.05, **P < 0.01, ****P < 0.0001 (two‐tailed). (D) Pearson correlation coefficients (Pearson's *r*) in wild‐type p53 cell lines (*n* = 72) for the gene effect scores for the indicated genes and the gene effect scores for *p53*. All significant correlations are indicated, *P < 0.05, **P < 0.01, ****P < 0.0001 (two‐tailed).

Wild‐type p53 function is frequently lost or impaired during cancer development by mutation. The DepMap portal data explorer tool was used to assess the association of gene effect scores for *S5A* and *ADRM1* with *p53* mutational status in Wellcome Sanger Institute whole‐genome CRISPR cancer cell line screens (Project Score) [54, 59, 60, 63, 81]. In support of the validity of the analysis, genes that express components of the ubiquitin system that repress the wild‐type p53 pathway, including *MDM2*, the deubiquitinating enzyme *HAUSP*/*USP7* [82] and the E2 ubiquitin‐conjugating enzyme *UBCH5C/UBE2D3* [83], showed a clear relationship between a smaller negative gene effect score and *p53* mutation (Fig. 7A,B). As would be anticipated, this indicates that loss of p53 function is linked with a reduction in the impact on cancer cell line viability of targeting these genes. For *S5A*, the Pearson's correlation coefficient (*r*) indicated, at best, a weak association between a smaller negative gene effect score and *p53* mutation, which just failed to reach significance (*r* = 0.10, *P* = 0.07; Fig. 7A,B). Interestingly, there was a significant correlation for *ADRM1* (*r* = 0.22, *P* = 0.00007), but *ADRM1* gene effect scores were low in this screen. Gene effect scores for other proteasome subunits were not significantly associated with p53 mutation or were significantly more negative in cell lines with mutant *p53* (Fig. 7B). This analysis suggests some tendency towards differences in the relationship between the *p53* mutational status and impact on cell viability of targeting S5A or ADRM1 compared with other proteasomal subunits.

The DepMap data explorer tool was also used to investigate the association between sensitivity to growth inhibition by wild‐type p53 and the effects of targeting S5A or ADRM1 on viability in Wellcome Sanger Institute knockout screens [54, 55, 56, 57, 58, 59, 60, 63, 81]. Consistent with its tumour suppressive role, gene effects scores for wild‐type *p53* were frequently positive, indicating increased cell growth on p53 knockout (Fig. 7C). As for *MDM2, HAUSP* and *UBCH5C*, the gene effect scores for *S5A* (*r* = −0.31, *P* = 0.008) were significantly negatively correlated with those for wild‐type *p53* (Fig. 7C,D). This indicates a bigger decrease in viability on targeting these genes in cell lines where wild‐type p53 is more growth limiting. A possible explanation is that upregulation of p53 following the targeting of a repressor has a larger impact on viability in these cell lines. There was also a significant association between the more modest gene effect scores for *ADRM1* and the scores for wild‐type *p53* (*r* = −0.24, *P* = 0.04). *S5A* and *ADRM1* were unique amongst proteasome subunit expressing genes in significantly exhibiting this negative correlation (Fig. 7D).

## Discussion

p53 is degraded by a predominantly S5A‐dependent pathway. At most, there is a minor dependency on ADRM1 and RAD23B in cells depleted of S5A. The degradation of p53 is markedly inhibited by siRNA‐mediated knockdown of S5A and by ectopic expression of S5AΔUIM, a deletion mutant of S5A lacking its UIMs that is incorporated into the proteasome. In contrast, the ubiquitin receptors S5A and ADRM1 and, to some extent, RAD23A and B play redundant roles in the degradation of MDM2. This indicates there are multiple non‐dependent pathways for the recruitment of MDM2 to the proteasome. Consistent with an independent pathway for RAD23 proteins, while the association of UBL‐UBA adaptors with the proteasome is greatly reduced in S5A and ADRM1 double‐knockout mice, there appears to be some residual binding activity [17]. Ectopic expression of S5AΔUIM combined with depletion of ADRM1 stabilised MDM2 indicating the UIMs of S5A participate in MDM2 degradation. This is consistent with the observation that S5A can bind to ubiquitinated MDM2 [15]. Ubiquitin receptor redundancy, as observed for MDM2, appears to be relatively common. S5A and ADRM1 were substantially redundant for the degradation of all other substrates examined in this study. Redundancy between these receptors has also been observed for limiting levels of NRF1 and beta‐catenin/CTNNB1 in mouse liver [17]. Furthermore, as reported for mouse liver and HEK293T cells [17], we observed that simultaneous targeting of S5A and ADRM1 caused a greater accumulation of ubiquitinated proteins than targeting either receptor alone. Individual suppression of S5A caused an intermediate increase in the level of ubiquitin conjugates, confirming that it can, at least partially, interfere with the degradation of substrates in addition to p53. It was recently reported that S5A binds to PTEN and depletion of S5A can increase PTEN stability resulting in AKT inhibition [84]. Studies in yeast also show a subset of ubiquitinated proteins are increased in level when Rpn10 alone is targeted [85, 86]. Proteome analysis and ubiquitin site profiling in human cells could be used to identify additional S5A‐dependent substrates [87]. This would enable investigation of the extent to which the accumulation of ubiquitin conjugates following S5A suppression is due to substantial stabilisation of a subset of substrates as opposed to minor effects on a broader range of ubiquitinated proteins.

Properties of p53 and MDM2 could influence their ubiquitin receptor dependency for proteasomal degradation. Both p53 and MDM2 have been reported to interact with subunits of the proteasome [88, 89, 90, 91], which could contribute to determining their pathways of degradation. The efficient proteasomal degradation of proteins requires an unstructured/disordered region at the N or C terminus where proteolysis is initiated [11]. There are unstructured regions at the termini of both p53 and MDM2 [92, 93]. The length, amino acid composition and distance from the site of ubiquitination of the unstructured regions in proteins impact their rate of degradation [94, 95]. It is possible that characteristics of the unstructured regions could influence the pathways through which a substrate is recruited to the proteasome. For example, it has been suggested that the distance between the unstructured region and the site of ubiquitination can better correspond to the distance in the proteasome between a particular ubiquitin receptor and the proteolysis initiation site [94]. There is an elegantly complex array of ubiquitin modifications. At one or more sites, proteins can be monoubiquitinated or conjugated to chains of ubiquitin of variable length involving links between any of the seven internal ubiquitin lysine residues or the N‐terminal methionine residue of ubiquitin [1]. Differences in the nature of p53 and MDM2 modification by ubiquitin could underlie the divergence in receptor dependency. The most prominent p53 conjugates accumulated following S5A knockdown formed a distinct ladder with electrophoretic mobility indicating one to six ubiquitin molecules per p53 molecule. In contrast, the knockdown of the ubiquitin receptors involved in MDM2 degradation generated a high molecular weight smear of MDM2 conjugates. A recent study based on *in vitro* analysis of the degradation of defined substrates by yeast proteasomes deficient in combinations of ubiquitin receptors provides evidence that the pattern of substrate ubiquitination can influence ubiquitin receptor preferences [30]. This indicated Rpn10 plays the major role in directly mediating the degradation of proteins modified with a single‐lysine 48‐linked ubiquitin chain, while Rpn10 and Rpn13 independently facilitate the degradation of proteins with multiple‐lysine 48‐linked ubiquitin chains. There are structural differences between yeast and human ubiquitin receptors, and it remains to be determined whether the orthologous receptors have the same activity in human proteasomes [9]. It would be of interest to characterise the p53 and MDM2 ubiquitin conjugates that accumulate following receptor suppression. In addition, evaluation of the effects of targeting ubiquitin receptors on global ubiquitin chain linkages and ubiquitin chain lengths would contribute to determining the relationships between the ubiquitin tag and the dependency on pathways of proteasomal recruitment for substrates in general [87, 96]. Comparison of the characteristics of sets of substrates, identified by proteome analysis and ubiquitin site profiling [87], that are stabilised following the suppression of ubiquitin receptors could enable the identification of molecular features of substrates that influence receptor dependency.

Reliance on a single receptor may facilitate modulation of the degradation of a subset of substrates by receptor regulation, while receptor redundancy ensures the unperturbed degradation of remaining substrates. S5A‐inhibiting pathways or stresses could efficiently upregulate p53 activity by inhibiting p53 degradation without stabilising its repressor MDM2. Several mechanisms are known through which S5A can be regulated. S5A can be selectively downregulated by several pro‐apoptotic agents [97, 98, 99]. p53 stabilisation due to reduced S5A levels could consequently contribute to promoting cell death. Proteasome associated and extraproteasomal S5A can undergo predominantly mono‐ or di‐ubiquitination [16, 100, 101, 102, 103]. The role of S5A ubiquitination requires further investigation, but it is thought to inhibit substrate recognition and it can be modulated by some cellular stresses. S5A is also one of the proteasome subunits that is conjugated to the UBL molecule NEDD8 in response to proteotoxic stress [104]. The HPV protein E6 re‐directs the endogenous E3 ligase E6AP to ubiquitinate p53, and this is involved in the development of cervical and head and neck cancers [105, 106]. Intriguingly, E6AP has been shown to bind to the proteasome through S5A [107, 108, 109]. The precise role is unclear, but there is evidence suggesting E6AP enhances proteasome activity [107] and increases ubiquitin binding to the proteasome [109]. It is possible that regulation of E6AP could influence S5A‐mediated substrate degradation. E6AP has been observed to promote MDM2‐dependent p53 degradation [107] and, in the absence of HPV‐E6, its depletion or loss causes p53 accumulation in some but not all contexts [110, 111, 112, 113]. In support of a role of modulation of proteasome‐bound E6AP, catalytically inactive mutants of E6AP can associate with S5A and have been reported to inhibit MDM2‐induced degradation of p53 [107].

High sensitivity of degradation to suppressing S5A alone could enable substrates such as p53 to participate in sensing the cellular demand for protein degradation. Cancer development, for example, is associated with an increase in the level of unfolded/misfolded proteins due to high translation rates, the accumulation of mutations, chromosomal abnormalities and environmental stresses such as UV exposure. A major route for the disposal of these proteins is ubiquitin‐dependent proteasomal degradation [114]. An increase in the cellular load of ubiquitinated unfolded proteins could cause p53 stabilisation by competing with p53 for S5A binding. p53 is upregulated by misfolded protein expression, and in addition to its anti‐proliferative and pro‐apoptotic roles, p53 can regulate genes involved in proteostasis [115, 116, 117]. A similar mechanism may underlie p53 induction by interventions that increase cellular levels of unanchored/substrate‐free polyubiquitin chains, including suppression of the deubiquitinating enzyme USP5/IsoT [52, 118, 119, 120, 121]. We previously observed that such interventions also cause p53 stabilisation and the accumulation of ubiquitinated p53 without affecting the degradation of MDM2 [52]. This selective stabilisation of p53 could be mediated by the competition of unanchored polyubiquitin chains for binding to S5A [12, 122]. It is possible that free polyubiquitin chains are involved in feedback control of the proteasome and that p53 upregulation may be a consequence of this [52, 123, 124, 125, 126]. A source of unanchored polyubiquitin chains is en‐bloc substrate deubiquitination by the intrinsic 19S RP subunit PSMD14/hRpn11. This allows ubiquitin recycling and may facilitate substrate entry into the proteasome [73]. S5A could be the ubiquitin receptor preferentially affected because it is in uniquely close proximity to PSMD14 in the substrate‐bound proteasome and is consequently near to the site of en‐bloc ubiquitin chain cleavage and PSMD14 can provide additional ubiquitin‐binding activity [11, 19, 127]. Strikingly, in yeast proteasomes, there is evidence that the affinity for lysine 48‐linked free polyubiquitin chain binding is far higher for Rpn10 than Rpn13. The inhibition constant/Ki of free chains of four ubiquitin molecules for Rpn10 was found to be over 100‐fold lower than that for Rpn13 [30]. Much remains to be determined regarding how free polyubiquitin chain levels are regulated, including the influence of stress and signalling pathways and the diverse cellular roles of different free polyubiquitin chains [128, 129, 130, 131, 132].

The data suggest that wild‐type p53 contributes to the reduction in viability of cancer cell lines resulting from the suppression of S5A but show that targeting S5A also has wild‐type p53‐independent effects. In HCT116 cells, the decrease in viability resulting from the suppression of S5A was partially attenuated by the loss of full‐length wild‐type p53 [15]. Furthermore, an assessment of Wellcome Sanger Institute CRISPR screens indicated that S5A knockout caused a larger decrease in viability in cell lines where wild‐type p53 has a greater growth‐limiting effect. This is consistent with the upregulation of p53 due to the targeting of S5A having a bigger impact on these lines. This relationship was not generally observed for 19S RP or 20S CP subunits. Similarly, the reduction in HCT116 cell viability due to the combined knockdown of S5A and ADRM1 or inhibition of the proteolytic activity of the proteasome by bortezomib was not attenuated by loss of full‐length p53. This is likely to be a result of the stabilisation of a broader range of proteasomal substrates, including MDM2 [15], and indicates mechanistic differences underlying the effects on cell viability of suppressing S5A and other ways of targeting the proteasome. Wild‐type p53 function is frequently lost during tumour development by *p53* mutation. In Wellcome Sanger Institute knockout screens, there was, at most, a weak correlation between the *p53* mutational status and the magnitude of the reduction in cell line viability caused by targeting S5A. This shows additional pathways are also involved. It has been reported that inhibition of AKT through stabilisation of PTEN can contribute to the effects of S5A depletion [84]. Inhibition of NF‐kappa B may also participate [133]. In the Wellcome Sanger Institute CRISPR screens, viability was assessed at 14 days: a reasonably extended time after virus transduction of the sgRNA library. The role of wild‐type p53 and its relative impact on viability may depend on the duration and magnitude of S5A suppression. In some contexts, wild‐type p53 activation through modulation of S5A could be involved in additional pathways such as protective adaptation to increases in the level of unfolded proteins or increased flux through the proteasome [115, 116]. Intriguingly, there were significant correlations in DepMap screens between the generally more modest effects on cell line viability of ADRM1 suppression and both *p53* mutational status and growth sensitivity to p53 loss. This supports the possibility that ADRM1 may also have an independent function in modulating the p53 pathway. In our study, there was a tendency towards a slight increase in p53 levels in cells depleted of ADRM1, but this did not achieve statistical significance and we did not detect p53 stabilisation. Alternative mechanisms could involve effects on one or more p53 pathway regulators or p53 target genes.

Different ways of interfering with proteasome function, such as targeting ubiquitin receptors, might overcome some mechanisms of tumour resistance to inhibitors of the proteolytic activities of the proteasome. Furthermore, targeting ubiquitin receptors would result in selective substrate stabilisation, at least in part due to ubiquitin receptor redundancy. This might have improved therapeutic efficacy compared with the currently used proteasome inhibitors because of differences in the balance of effects on cellular pathways. Targeting several ubiquitinated‐substrate shuttle proteins, most notably UBQLN2, UBQLN4 and DDI2, impacted viability in DepMap screens (Fig. S4). We observed relatively modest effects on cell line viability of ADRM1 knockdown. In DepMap screens, negative gene effect scores for *ADRM1* were also frequently small. However, in CRISPR screens, *ADRM1* was classified as essential in around 20% of cell lines. Small molecule inhibitors of ADRM1 are being developed for the treatment of cancer [134, 135, 136, 137]. S5A is also an attractive potential target for cancer therapy. Depleting S5A alone substantially reduced the viability of the cancer cell lines tested in this study. Furthermore, knockdown of S5A has been reported to reduce the viability of several additional lines [43, 84, 133, 138]. DepMap dependency probability analysis classified *S5A* as essential in half of cancer cell lines in RNAi screens, and this increased to virtually all lines in CRISPR screens [54, 62]. S5A could be targeted for therapy by interventions that reduce its levels, interfere with its association with the proteasome, competitively or allosterically inhibit its binding to ubiquitin or prevent changes in conformation required for substrate degradation [135, 139, 140]. As there may be variations in the consequences of targeting S5A in different ways, [34, 74, 76, 86, 141] it will be important to use CRISPR in normal and cancer cells to compare the therapeutic potential of S5A loss and the targeting of individual domains, particularly the UIMs of S5A. It would also be of interest to carry out global proteome and ubiquitome analysis and gene expression profiling to identify additional pathways that participate in the effects on cell viability of S5A suppression.

## Author contributions

MKS conceived and supervised the study; AS, CJK and MKS designed and carried out experiments, analysed data and prepared figures; MKS wrote the manuscript.

## Supporting information


**Fig. S1.** siRNAs used for the experiments shown in Fig. S2.

**Fig. S2.** Targeting a range of ubiquitin receptors did not substantially influence p53 or MDM2 stability.

**Fig. S3.** Confirmation of the importance of the proteasome in A375 cells.

**Fig. S4.** Targeting some ubiquitinated‐substrate shuttle proteins impacts the viability of cancer cell lines.

## Data Availability

The data that support the findings of this study are openly available in figshare at https://doi.org/10.6084/m9.figshare.6025238.v6, https://doi.org/10.6084/m9.figshare.14461980.v2 and https://doi.org/10.6084/m9.figshare.19139906.v1.
