# Supplementary figures and images for: Ubiquitin receptors play redundant roles in the proteasomal degradation of the p53 repressor MDM2
Source: FEBS Lett. 2022 Jul 21;596(21):2746–67. doi: 10.1002/1873-3468.14436 (PMC9796813; doi:10.1002/1873-3468.14436)

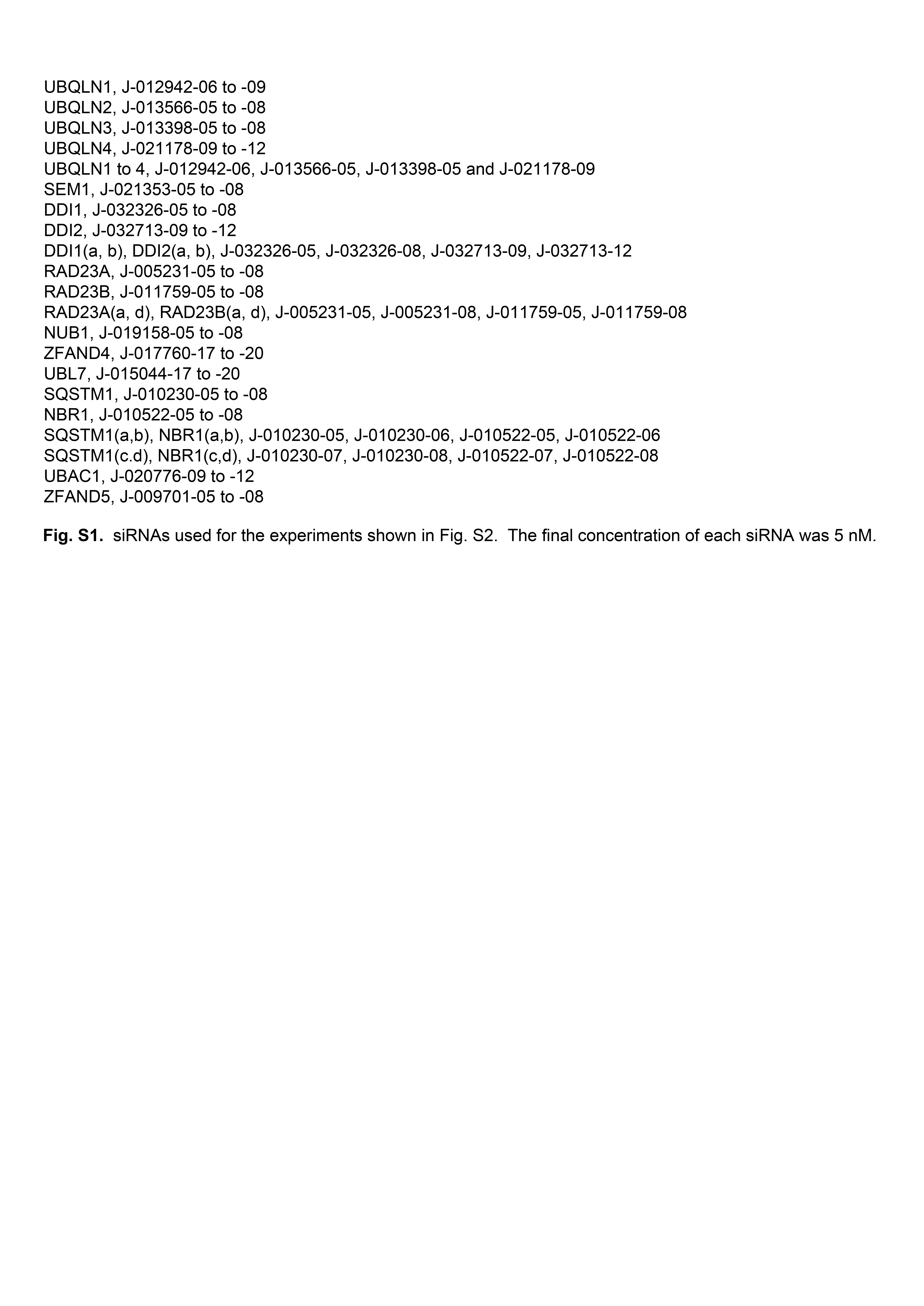

Supplement: Supplementary file 1 — Fig. S1. siRNAs used for the experiments shown in Fig. S2. [file FEB2-596-2746-s002.tif]

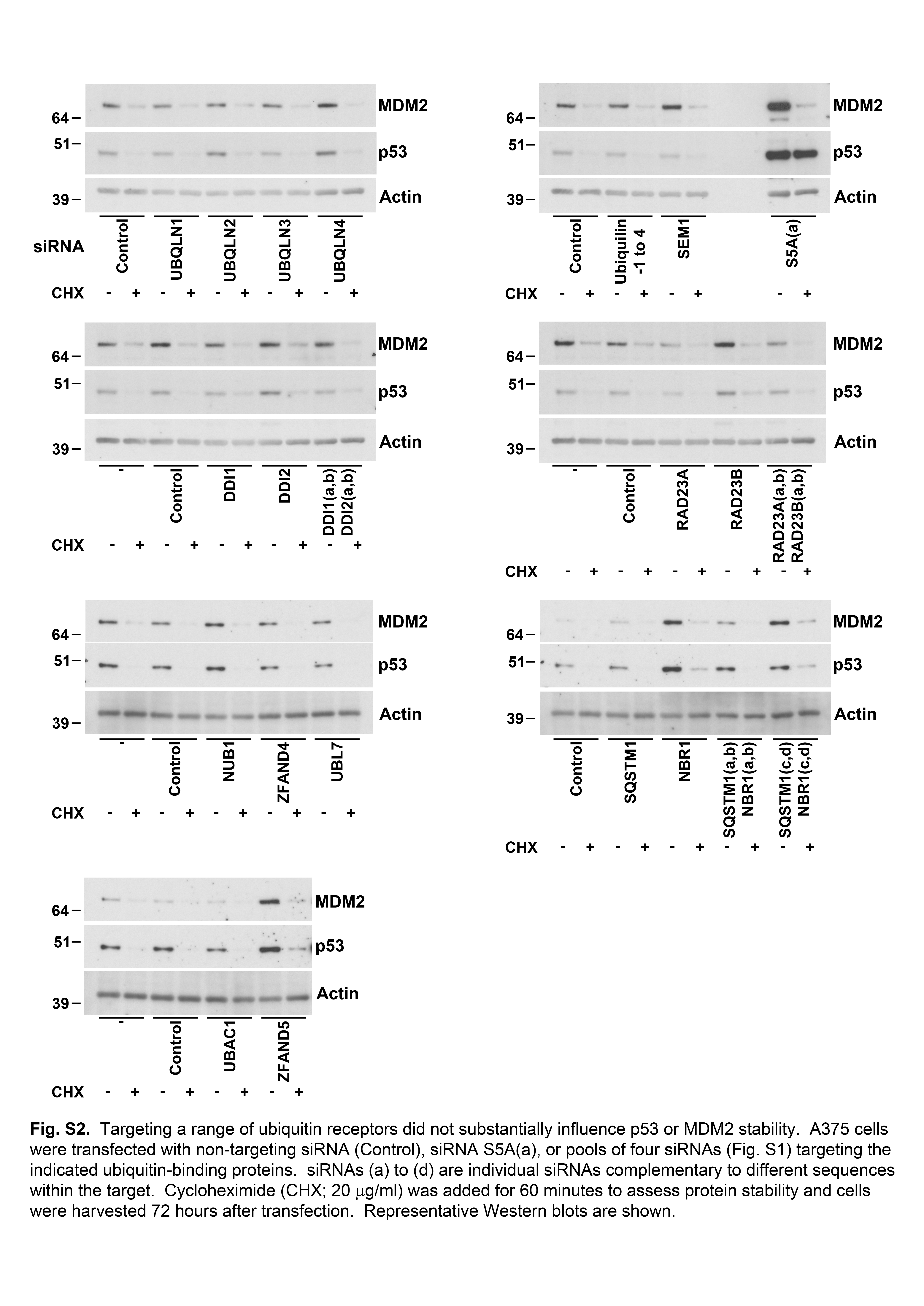

Supplement: Supplementary file 2 — Fig. S2. Targeting a range of ubiquitin receptors did not substantially influence p53 or MDM2 stability. [file FEB2-596-2746-s004.tif]

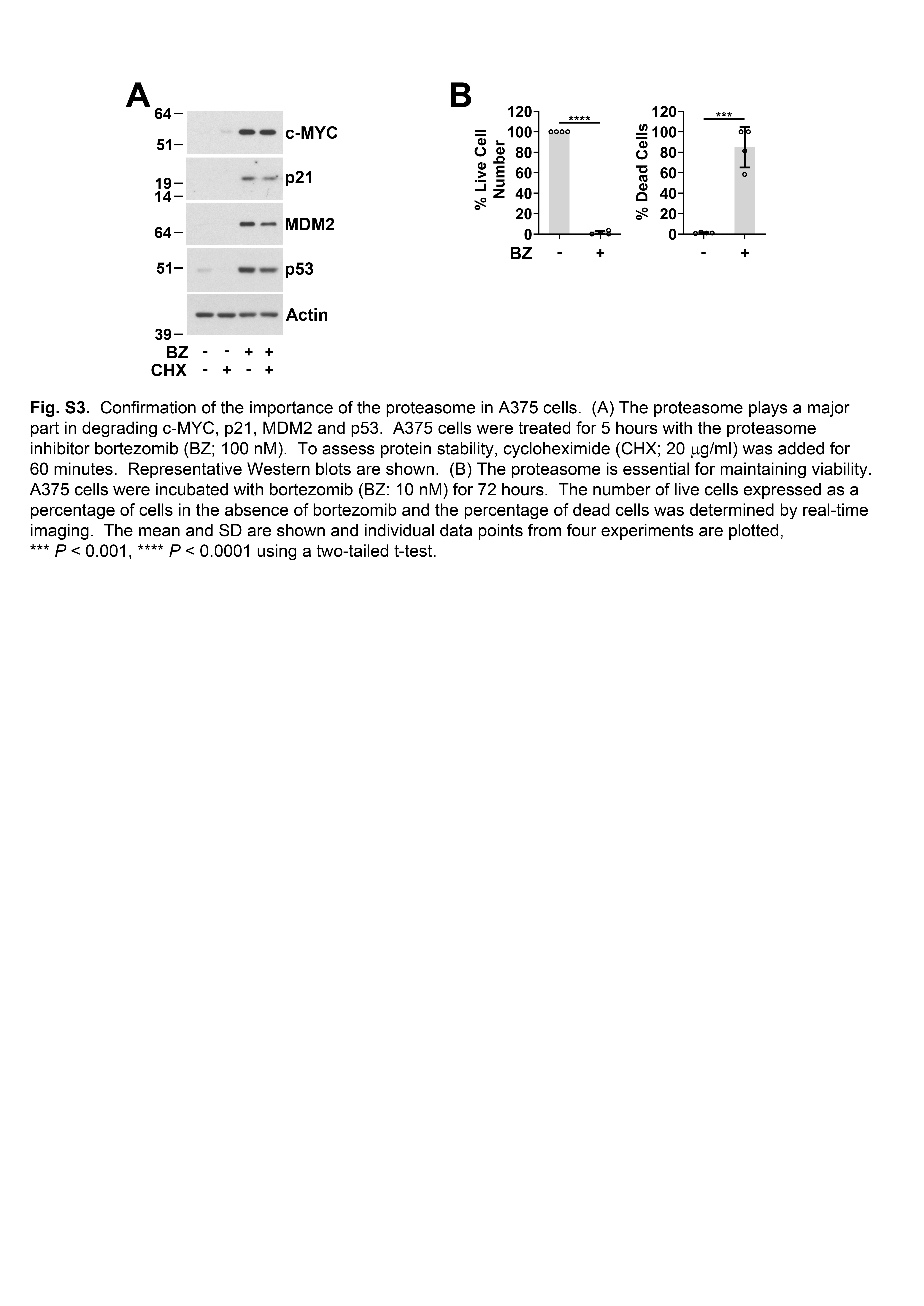

Supplement: Supplementary file 3 — Fig. S3. Confirmation of the importance of the proteasome in A375 cells. [file FEB2-596-2746-s001.tif]

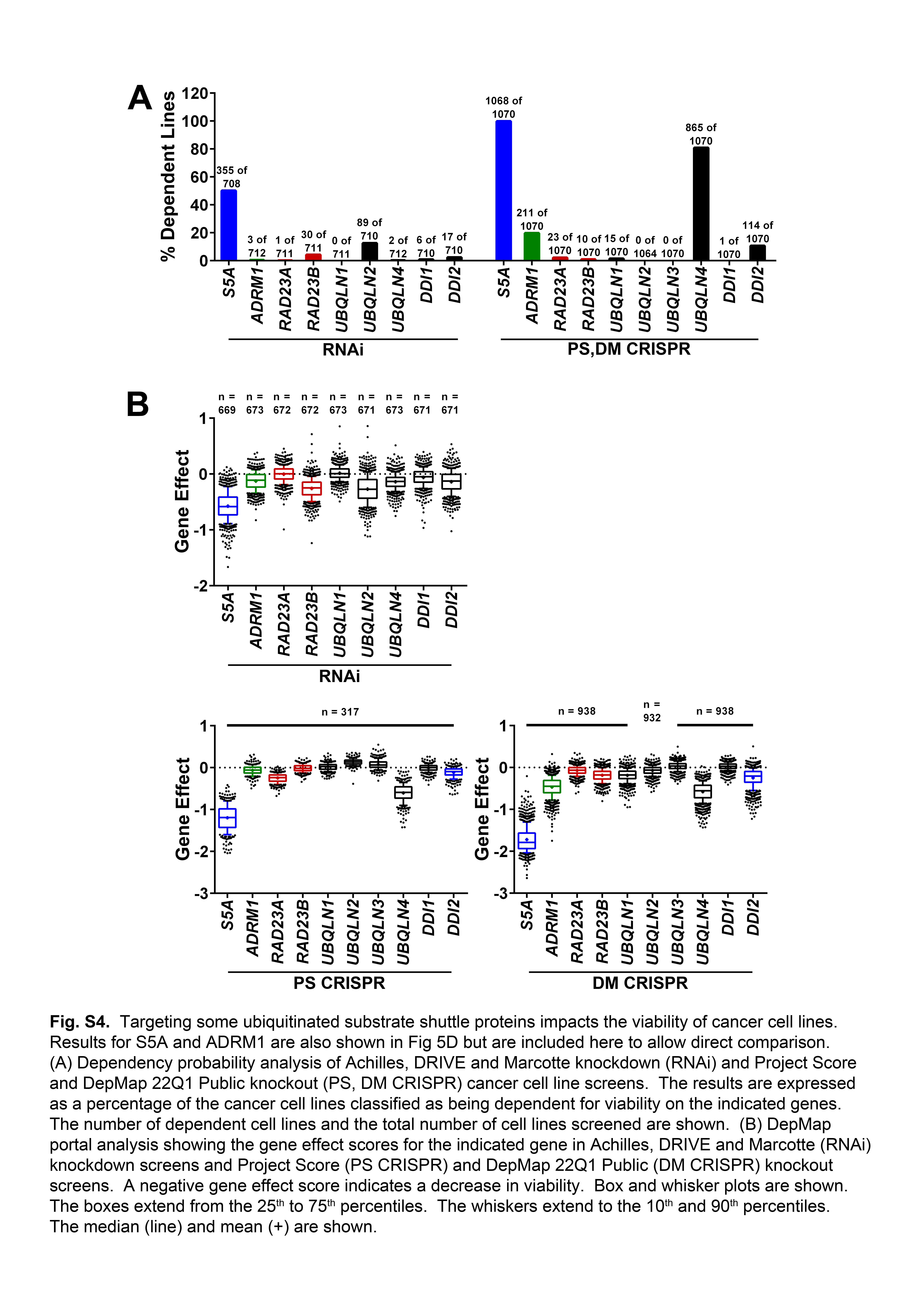

Supplement: Supplementary file 4 — Fig. S4. Targeting some ubiquitinated‐substrate shuttle proteins impacts the viability of cancer cell lines. [file FEB2-596-2746-s003.tif]
